# Tongbian decoction restores intestinal microbiota and activates 5-hydroxytryptamine signaling: implication in slow transit constipation

**DOI:** 10.3389/fmicb.2023.1296163

**Published:** 2024-01-15

**Authors:** Hongjia Li, Na Lv, Dongna Li, Yunzhi Qian, Xianghuan Si, Yuanqing Hua, Yujuan Wang, Xiaojuan Han, Tianshu Xu

**Affiliations:** ^1^Department of Traditional Chinese Medicine, Nanjing Drum Tower Hospital, The Drum Tower Clinical Medical College, Nanjing University of Chinese Medicine, Nanjing, China; ^2^Department of Nutrition, Gillings School of Global Public Health, University of North Carolina at Chapel Hill, Chapel Hill, NC, United States; ^3^Department of Traditional Chinese Medicine, Nanjing Drum Tower Hospital, Affiliated Hospital of Medical School, Nanjing University, Nanjing, China; ^4^Department of Rheumatology and Immunology, Nanjing Drum Tower Hospital, Affiliated Hospital of Medical School, Nanjing University, Nanjing, China

**Keywords:** slow transit constipation, tongbian decoction, intestinal microbiota, 5-hydroxytryptamine, fecal microbiota transplant

## Abstract

**Introduction:**

Slow transit constipation (STC) is a type of functional constipation. The detailed mechanism of STC, for which there is currently no effective treatment, is unknown as of yet. Tongbian decoction (TBD), a traditional Chinese medicinal formula, is commonly used to treat STC in clinical settings. However, the potential impact of TBD on the management of STC via modulation of the gut microbiota remains unclear.

**Methods:**

Pseudo-germ-free rats were constructed after 6 days of treatment with bacitracin, neomycin, and streptomycin (abbreviated as ABX forthwith). Based on the successful construction of pseudo-germ-free rats, the STC model (ABX + STC) was induced using loperamide hydrochloride. After successful modeling, based on the different sources of donor rat microbiota, the ABX + STC rats were randomly divided into three groups: Control → ABX + STC, STC → ABX + STC, and STC + TBD → ABX + STC for fecal microbiota transplant (FMT). Body weight, fecal water content, and charcoal power propelling rate of the rats were recorded. Intestinal microbiota was detected by 16S rRNA sequencing, and the 5-hydroxytryptamine (5-HT) signaling pathway was examined by western blots, immunofluorescence, and immunohistochemical analysis.

**Results:**

After treatment with fecal bacterial solutions derived from rats treated with Tongbian decoction (TBD), there was an increase in body weight, fecal water content, and the rate of charcoal propulsion in the rats. Additionally, activation of the 5-hydroxytryptamine (5-HT) signaling pathway was observed. The 16S rRNA sequencing results showed that the fecal bacterial solution from TBD-treated rats affected the intestinal microbiota of STC rats by increasing the proliferation of beneficial bacteria and suppressing the expansion of harmful bacteria.

**Conclusion:**

Our study showed that TBD alleviated constipation in STC rats by modulating the structure of the intestinal microbiota.

## 1 Introduction

Slow transit constipation (STC) is caused by colon transport dysfunction and conduction abnormalities. Its clinical manifestations are often characterized by longer defecation time and difficulty in defecation, usually without obvious organic lesions, which can seriously affect the quality of life of patients (Inoue et al., 2017; Patton et al., 2020; Wang L. et al., 2022). The number of patients with STC in China is increasing annually, and the prevalence rate of STC is approximately 3.19–11.6% (Bharucha and Lacy, 2020; Yao et al., 2022). Various factors such as Cajal’s interstitial cells, immune factors, endocrine factors, laxatives, psychological factors, diet, and exercise habits have been implicated in the onset and exacerbation of STC (He et al., 2000; Lyford et al., 2002; Wedel et al., 2002; Foong et al., 2020; Serra et al., 2020; Wang H. et al., 2022; Kaji and Hori, 2023). An increasing number of studies have shown that gut microbiota and neurotransmitters are closely related to STC (Ding et al., 2018; Ohkusa et al., 2019; Göthert et al., 2020).

Intestinal microbiota resides in different parts of the small and large intestines, influencing host material metabolism, nutrient absorption, and gastrointestinal motions (Clemente et al., 2012; Simrén et al., 2013). Intestinal microbial dysbiosis can cause constipation (Yoo and Mazmanian, 2017; Ohkusa et al., 2019). A previous study has found differences in the relative abundance of species and alpha diversity between patients with STC and healthy subjects (Guo et al., 2020). Therefore, the gut microbiota may be a potential therapeutic target for STC.

Currently, the clinical treatment of STC includes pharmacological and non-pharmacological approaches (Kassim et al., 2020). Common drug-based treatments typically feature laxatives and 5-hydroxytryptamine (5-HT) agonists (Tsukamoto et al., 2011; Hoffman et al., 2012). However, discontinuation of these treatments often leads to recurrence. Therefore, the development of safer and more effective treatment strategies for STC is imperative. Traditional Chinese medicines, composed of natural ingredients, work through multiple targets and pathways (Lin et al., 2009; Wang et al., 2012; Li et al., 2017). Upon entering the human body, these medicines interact with intestinal microbiota, impacting their absorption, distribution, and metabolism to achieve therapeutic effects (Li et al., 2017; Zeyue et al., 2022; Zhao et al., 2022).

In the past few decades, Tongbian decoction (TBD) has been proven effective in treating STC in clinical practice (Tianshu, 2012). TBD are compound medicine composed of twelve Chinese herbs, including *Scrophulariae radix*, *Rehmanniae radix* preparate, *Armeniacae semen* amarum, *Atractylodes macrocephala* rhizome, *Aurantii fructus*, *Trichosanthis semen*, *Cannabis fructus*, *Pruni semen*. Pharmacology analysis revealed that it contains main active ingredients, including kaempferol, quercetin, luteolin, naringenin, (+)-catechin, neohesperidin, etc.

TBD—a traditional Chinese medicine that nourishes the Yin, aids defecation, regulates qi, and distributes jin—has demonstrated promising outcomes in the clinical treatment of STC (Tianshu, 2012). A previous study has shown that TBD can alleviate constipation symptoms and increase bowel movements in patients (Rongzhi, 2014). The clinical experimental study shows that the clinical efficacy of TBD in the treatment of slow transit constipation is significantly better than that of mosapride citrate dispersible tablets (Tianli, 2020). Animal experimental studies have shown that TBD can alleviate STC by regulating intestinal SP content and down-regulating intestinal VIP content (Haixia et al., 2021). Moreover, compared with Western medicine, TBD has the advantages of fewer side effects, lower recurrence rates, and significant therapeutic efficacy. In addition, our previous study confirmed that TBD can regulate the gut microbiota environment in patients with STC (Shuai et al., 2017). Although the efficacy of TBD for STC has been established, it is still not clear whether its therapeutic action is mediated through regulation of the gut microbiota.

In this study, we transferred the gut microbiota of TBD-treated rats to rats with STC to explore whether the intestinal microbiota is a potential therapeutic target through which TBD alleviates STC symptoms.

## 2 Materials and methods

### 2.1 Composition of TBD

Tongbian decoction were purchased from Nanjing Drum Tower Hospital Traditional Chinese Medicine pharmacy. Chinese medicinal materials was purchased from Haozhou Liushuntang Pharmaceutical Co., Ltd. (Anhui, China). Tongbian decoction is composed of 12 types of Chinese herbs such as *Denophorae radix*: 20 g (Nanshashen in Chinese), *Ophiopogonis radix*: 20 g (Maidong in Chinese), *Scrophulariae radix*: 30 g (Xuanshen in Chinese), *Rehmanniae radix* preparate: 30 g (Shudihuang in Chinese), *Armeniacae semen* amarum: 10 g (Xingren in Chinese), *Atractylodes macrocephala* rhizome: 40 g (Baizhu in Chinese), *Aurantii fructus*: 15 g (Zhiqiao in Chinese), *Magnolia officinalis* cortex: 10 g (Houpu in Chinese), *Trichosanthis semen*: 20 g (Gualouren in Chinese), *Cannabis fructus*: 10 g (Huomaren in Chinese), *Pruni semen*: 15 g (Yuliren in Chinese), and *Radix Aucklandiae* radix: 8 g (Muxiang in Chinese) were combined, at a total of 230 g. Tongbian decoction each 1 mL containing 3 g raw medicine, autoclaved in bottles for later use.

### 2.2 Animals

All male Sprague Dawley (SD) rats used in this study (210–230 g, 8 weeks old) were purchased from Beijing Si Pei Fu Biotechnology Co., Ltd. (Beijing, China) and maintained under a specific pathogen-free (SPF) facility. All rats were housed in cages with free access to food and water at a controlled temperature (23 ± 2°C), a humidity of 50–55%, and a 12-h light–dark cycle. All animal experiments are carried out in accordance with the ethical principles of animal experiments in the Declaration of Helsinki. It was approved by the Laboratory Animal Ethics Committee of Nanjing Hospital Affiliated with Nanjing Medical University (approval number: DWSY-22113242).

### 2.3 Preparation of fecal bacterial solution

After 1 week of adaptive feeding, nine donor rats were average divided into three groups: Control, STC, and STC + TBD. The STC and STC + TBD groups were treated with 3 mg/kg/d loperamide hydrochloride (No. LLJO007, Xi’anJansen Co., Ltd.) from day1 to day7 to construct an STC model (Ren et al., 2017). During days 8 to 21, the model was maintained for the STC group, whereas the STC + TBD group was additionally treated with 24 g/kg TBD. The Control group was administered the same volume of physiological saline by oral gavage. One week after the administration of TBD, fresh fecal samples from the Control, the STC, and the STC + TBD groups were collected on a clean bench, mixed with sterile phosphate-buffered saline (PBS; fecal weight to sterile PBS volume ratio, 1:5), and immediately vortexed. After stool collection on day 21, all rats were sacrificed under isoflurane anesthesia. A porous nylon filter was used to remove large particles and fibrous substances to prepare the fecal suspension (Sun et al., 2020).

### 2.4 Preparation of animal models

Eighteen recipient rats were treated with 200 mg/kg of the antibiotics: bacitracin (No. B8181, Solarbio), neomycin (No. N8090, Solarbio), and streptomycin (No. S8290, Solarbio) (abbreviated as ABX forthwith). ABX were administered for 6 days to eliminate intestinal microbiota and construct a pseudo-germ-free rat model (Wichmann et al., 2013; Fan et al., 2018; Liao et al., 2021). To confirm the successful creation of this model, 16S rRNA sequencing was performed on rats that had been treated with the broad-spectrum ABX.

Based on pseudo-germ-free rats, the STC model was established by administering loperamide hydrochloride (ABX + STC) for 1 week (Schuijt et al., 2016; Zeyue et al., 2022). After successful modeling, based on the different sources of donor rat microbiota, the ABX + STC rats were randomly divided into three groups: Control → ABX + STC, STC → ABX + STC, and STC + TBD → ABX + STC group. The fecal suspension of donor microflora was transplanted into recipient rats by fecal microbiota transplantation (FMT) at a concentration of 1 mL/100 g body weight by intragastric administration over a 6-day period (Yano et al., 2015).

### 2.5 Measurement of fecal water content

On the 1 st, 6 th, 13 th, and 19 th days, the feces of the rats in each group were collected and wet weights were measured. The feces were then placed into an electric thermostatic air-drying oven (Shanghai Jinghong Experimental Equipment Co., Ltd.; Model: DHC-9030A) set at 70°C for overnight drying. The dry weight of the fecal matter was then obtained, and the fecal water content was calculated according to the following equation: (wet weight − dry weight)/wet weight × 100% (Yao et al., 2022).

### 2.6 Measurement of the charcoal power propelling rate

After the rats were fasted for 12 h, all groups were administered a 5% activated carbon suspension gavage (10 mL/1 kg), which comprises a 5% activated carbon suspension configuration dissolved in 100 mL distilled water, along with 3 g gum arabic (Suzhou Yijing Food Additives Co., Ltd.), and 5 g kaolin (Tianjin Beilian Fine Chemicals Development Co., Ltd.). After the activated carbon suspension for 30 min, rats were immediately sacrificed with isoflurane anesthesia. The entire intestine was excised from the lower end of the pylorus to the upper anus. Both the total intestinal length and the length of the activated charcoal suspension in the intestine were measured. The intestinal transit rate was calculated as follows: distance of the activated carbon in the whole intestine (cm)/total length of the entire intestine (cm) × 100% (Wang X. et al., 2022).

### 2.7 Hematoxylin–eosin (HE) staining

Colon tissue was obtained from 2 cm below the cecum. Colon tissue was initially fixed with 4% paraformaldehyde, dehydrated, impregnated, embedded, sectioned into 5 mm thick blocks, dewaxed for transparency, and stained with HE (No. S5545, No. G1001, Servicebio). Subsequently, changes in colonic tissue were observed under a standard light microscope (DM500, Leica, Germany) (Shi et al., 2020). Three colon sections were selected from each group, and three microscope fields were randomly selected on each section. The histological scores were graded as follows: (1) inflammation severity (0 = none, 1 = mild, 2 = moderate, 3 = severe); (2) inflammation extent (0 = none, 1 = mucosa, 2 = submucosa, 3 = transmural); (3) crypt damage (0 = none, 1 = base 1/3 damage, 2 = base 2/3 damage, 3 = crypt lost but surface epithelium present, 4 = crypt and surface epithelium lost); and (4) proportion of tissue involvement (1 = 0–25%, 2 = 26–50%, 3 = 51–75%, 4 = 76–100%) (Dieleman et al., 1998; Lv et al., 2021).

### 2.8 Immunohistochemical staining

The expression of 5-HT in colonic tissues was analyzed using immunohistochemistry. Tissue slides were dewaxed with xylene, rehydrated and incubated in 100°C in citrate buffer pH 6 for 20 min for antigen retrieval, incubated in 3% H_2_O_2_ for 25 min to quench endogenous peroxidases, blocked with normal goat serum for 10 min, and incubated with a 5-HT primary antibody (1:5000, No. S5545, Sigma) at 4°C for 12 h, washed with PBS, incubated for 2 h at room temperature for secondary antibody (1:200, No. GB23303, Servicebio), and finally developed using the DAB horseradish peroxidase color development kit (No. G1212, Servicebio). After counterstaining with hematoxylin, the slides were mounted with neutral balsam and observed under a light microscope (DM500, Leica, Germany). The mean optical density of each visual field was measured using the ImageJ software (ImageJ-win64) (Schneider et al., 2012; Qu et al., 2021).

### 2.9 Immunofluorescence

Immunofluorescence co-localization analysis of the expression and distribution of intestinal chromaffin and 5-HT in colon epithelial cells was performed. Tissue sections were dewaxed with xylene, rehydrated and incubated in 100°C in citrate buffer pH 6 for 20 min for antigen retrieval, incubated in 3% H_2_O_2_ for 25 min to quench endogenous peroxidases, blocked with normal goat serum for 10 min, and incubated with a 5-HT primary antibody (1:5000, No. S5545, Sigma) at 4°C for 12 h, washed with PBS, incubated with a red fluorescent secondary antibody (1:500, No. A0516, Biyuntian) for 1 h at room temperature. Slides were washed 3 times with PBS, a Chromogranin A primary antibody (1:800, No. 60135, Proteintech) was added and incubated overnight at 4°C and an Alexa Fluor 488-tagged secondary antibody (1:500, No. A0428, Biyuntian) was added and incubated for 1 h at room temperature. Finally, nuclear staining was performed using DAPI (No. C0204002; Bioss). Images were captured using a fluorescence microscope (DMi8, Leica, Germany) (Yano et al., 2015; Cao et al., 2017).

### 2.10 Western blot analysis

The proximal colon tissue of the SD rats was pulverized, and total protein was extracted from the colon tissue using RIRA lysis buffer (No. G2002, Servicebio). The protein concentration was determined using a BCA kit (No. G2026, Servicebio), and the total protein suspension was run on 10% SDS-PAGE gels (No. G2037, Servicebio) and subsequently transferred to a PVDF membrane. The PVDF membrane was incubated with primary antibodies against tryptophan hydroxylase-1 (TPH1) (1:1000, No. 12339S, CST), 5-HT4 receptor (5-HT4R) (1:1000, No. A2802, ABclonal) and GAPDH antibody (1:2000, No. GB15004, Servicebio) overnight at 4°C, incubated with the secondary antibodies (1:1000, No. GB23303, Servicebio) at room temperature for 1 h, washed with TBST, and developed with an ECL luminescent solution. ImageJ software was used to analyze the gray values of the protein bands, using GAPDH as an internal reference (Zhan et al., 2022).

### 2.11 16S rRNA sequencing

Rat feces were collected and quickly stored in a −80°C ultra-low temperature refrigerator for 16S rRNA gene amplicon sequencing. Total genomic DNA was extracted from fecal bacteria using a DNA extraction kit (TianGen). Then, the V3-V4 region of the bacterial 16S rRNA gene in each sample was amplified with primers 515F (5′-GTGCCAGCMGCCGCGGTAA-3′) and 806R (5′-GGACTACHVGGGTWTCTAAT-3′). Sequencing libraries were generated using NEB Next^®^ Ultra^™^ II FS DNA PCR-free Library Prep Kit (New England Biolabs, USA, Catalog #: E7430L). The library was checked with Qubit and real-time PCR for quantification and bioanalyzer for size distribution detection. Quantified libraries were pooled and sequenced on Illumina platforms (Novogene, Beijing China), according to effective library concentration and data amount required. Paired-end reads were assigned to the samples according to the unique barcode and truncated by cutting off the barcode and primer sequence of the samples. Paired-end reads were merged using Fast Length Adjustment of SHort reads (FLASH V1.2.11) and clean tags were obtained using fastp (Version 0.23.1) quality control high-quality. The tags were then compared with the Silva database using the UCHIME Algorithm to remove chimera sequences and obtain effective tags. The QllME software was used to process the sequences and microbial composition analysis (Hu et al., 2022; Zhang et al., 2022).

### 2.12 Statistical analysis

All data are presented as the mean ± standard deviation. Graphpad prism 7 (Graphpad Software Inc., USA) and SPSS 19.0 (IBM SPSS software, USA) were used to analyze the data. Statistical differences between the two groups were determined using the Student’s *t*-test. Multiple groups were compared using one-way analysis of variance followed by Tukey’s multiple comparison test. Statistical significance was considered at *p* < 0.05.

## 3 Results

### 3.1 Broad-spectrum antibiotics promote intestinal peristalsis

**Figure 1:**
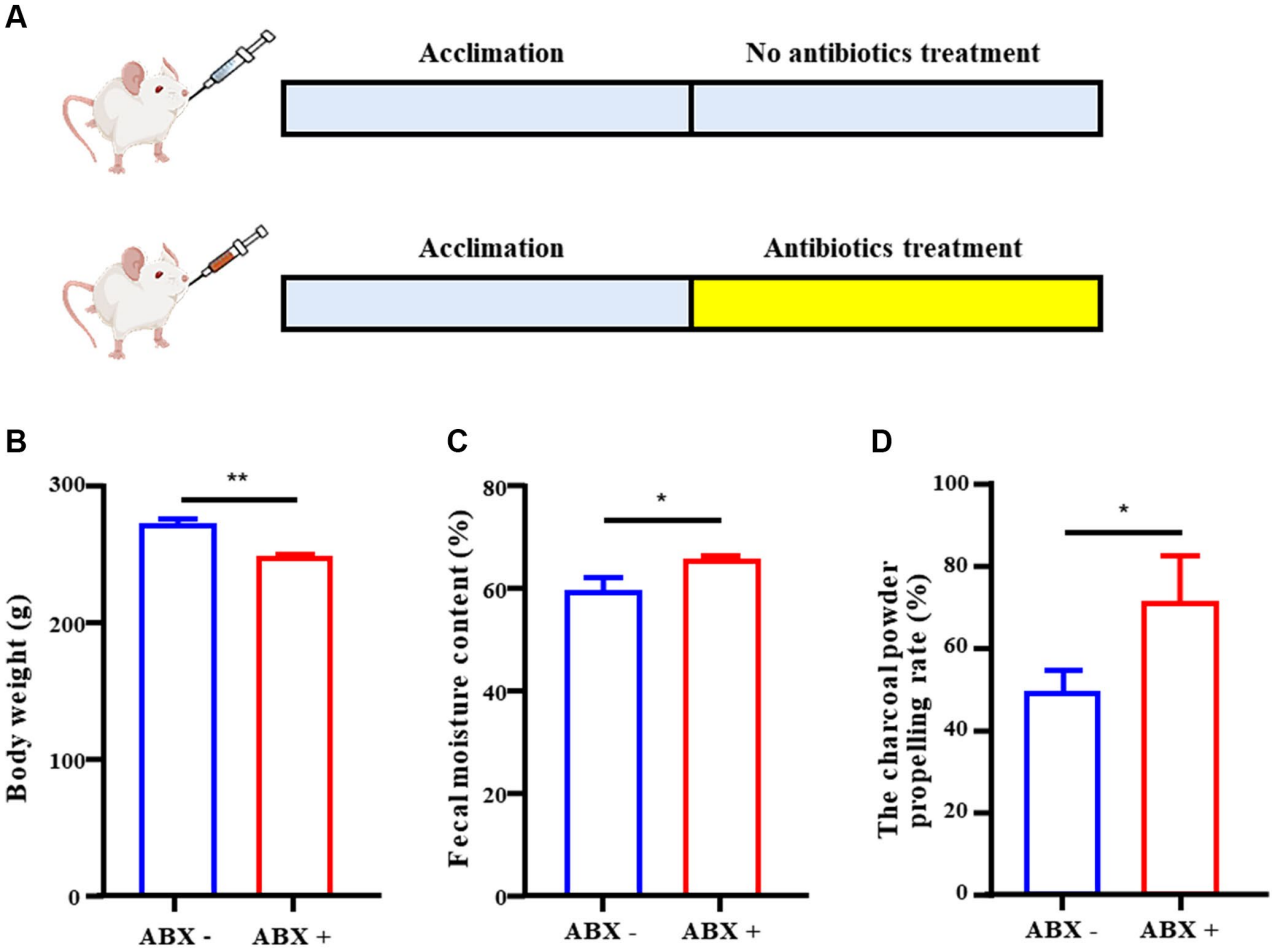
Influence of gut microbes on intestinal motility. **(A)** Design experiment. **(B)** Body weight of the ABX – and the ABX + group. **(C)** The fecal moisture content of the ABX – and the ABX + group. **(D)** The charcoal power propelling rate of the ABX – and the ABX + group. Data were represented as mean ± SD. ABX −: no antibiotics treatment, ABX +: antibiotics treatment. ^*^p < 0.05, ^**^p < 0.01 (comparison with ABX – group).

To understand the role of the intestinal microflora in intestinal motility, pseudo-germ-free rats (ABX+) were constructed (Figure 1A). The ABX + group showed a significant reduction in body weight (*p* < 0.01) (Figure 1B). Moreover, compared with the ABX - group, fecal water content and charcoal power propelling rate in the ABX + group were significantly higher (*p* < 0.05) (Figures 1C,D). These results suggested that intestinal microbiota plays a role in regulating intestinal motility in rats.

### 3.2 Broad-spectrum antibiotic treatment depletes the intestinal microflora

**Figure 2:**
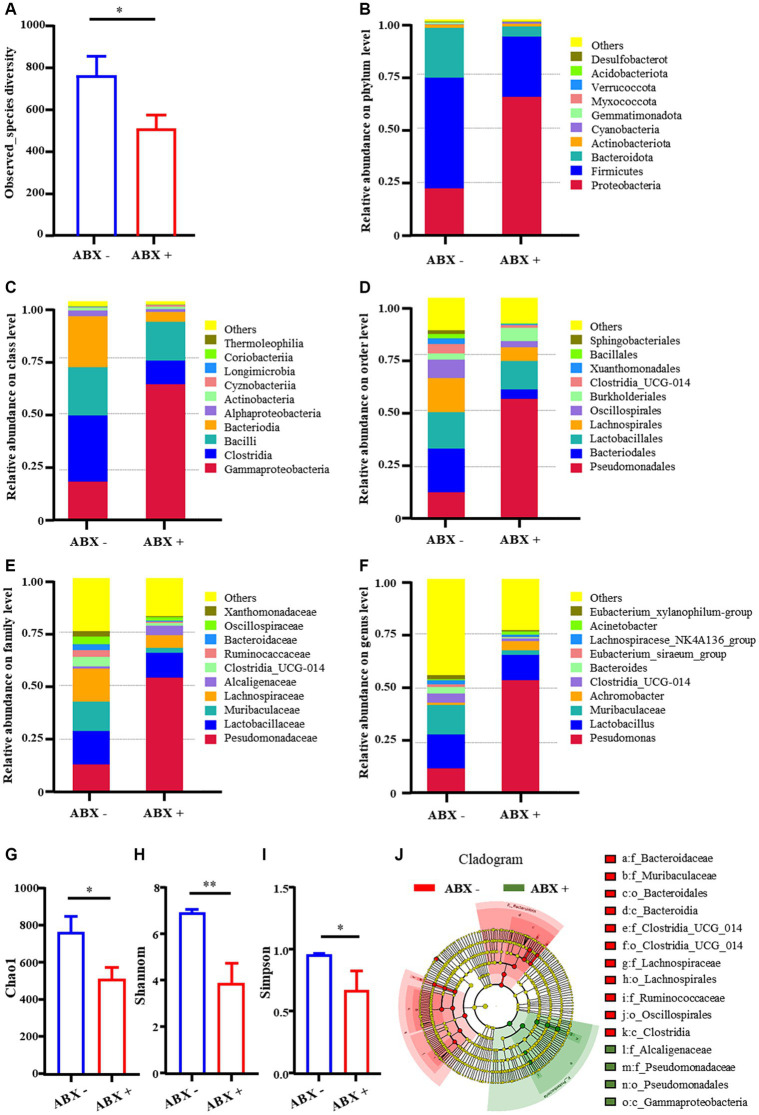
Relative abundance of intestinal microflora. **(A)** Observed_species diversity. **(B–F)** Phylum, Class, Order, Family, Genus levels. **(G–I)** Alpha-diversity of intestinal bacteria. **(J)** Cladogram showing the phylogenetic relationships of bacteria taxa. Data are expressed as the mean ± SD. ABX −: no antibiotics treatment, ABX +: antibiotics treatment. ^*^p < 0.05, ^**^p < 0.01 (comparison with ABX – group).

The findings in the previous paragraph suggest that the intestinal microbiota may affect the gut motility. Next, we explored whether intestinal microbiota is a key mechanism for TBD in alleviating constipation using a pseudo-germ-free rat model. 16S rRNA sequencing results revealed that the observed_species diversity of rate decreased significantly in the ABX + group (515.6 ± 65.5) compared with those ABX – group (768.6.0 ± 89.7) (Figure 2A). This highlights a transformation in the number of species. At various taxonomic levels—ranging from phylum, class, order, family to genus, the abundance of most intestinal microbiota was notably reduced (Figures 2B–F). The Chao1 were also significantly decreased in the ABX + group, indicating a significant decline in microbial abundance in the gut (Figure 2G). The Shannon and Simpson were also significantly decreased in the ABX + group, indicating a significant decline in community diversity in the gut (Figures 2H,I). To further clarify the differences in the diversity of the rat intestinal microbiota among the different treatment groups, linear discriminant analysis Effect Size (LEfSe) analysis was used to explore the effects of intestinal bacteria from phylum to genus. The special bacteria in the ABX- group were *Bacteroidaceae, Muribaculaceae, Clostridia_UCG_014, Lachnospiraceae, Oscillospirales*, etc. The specific bacteria in the ABX+ group were *Pseudomonadaceae, Alcaligenaceae*, and *Gammaproteobacteria* (Figure 2J). These results confirm that pseudo-germ-free rats were successfully prepared.

### 3.3 TBD fecal transplants alleviate STC phenotype in rat model

**Figure 3:**
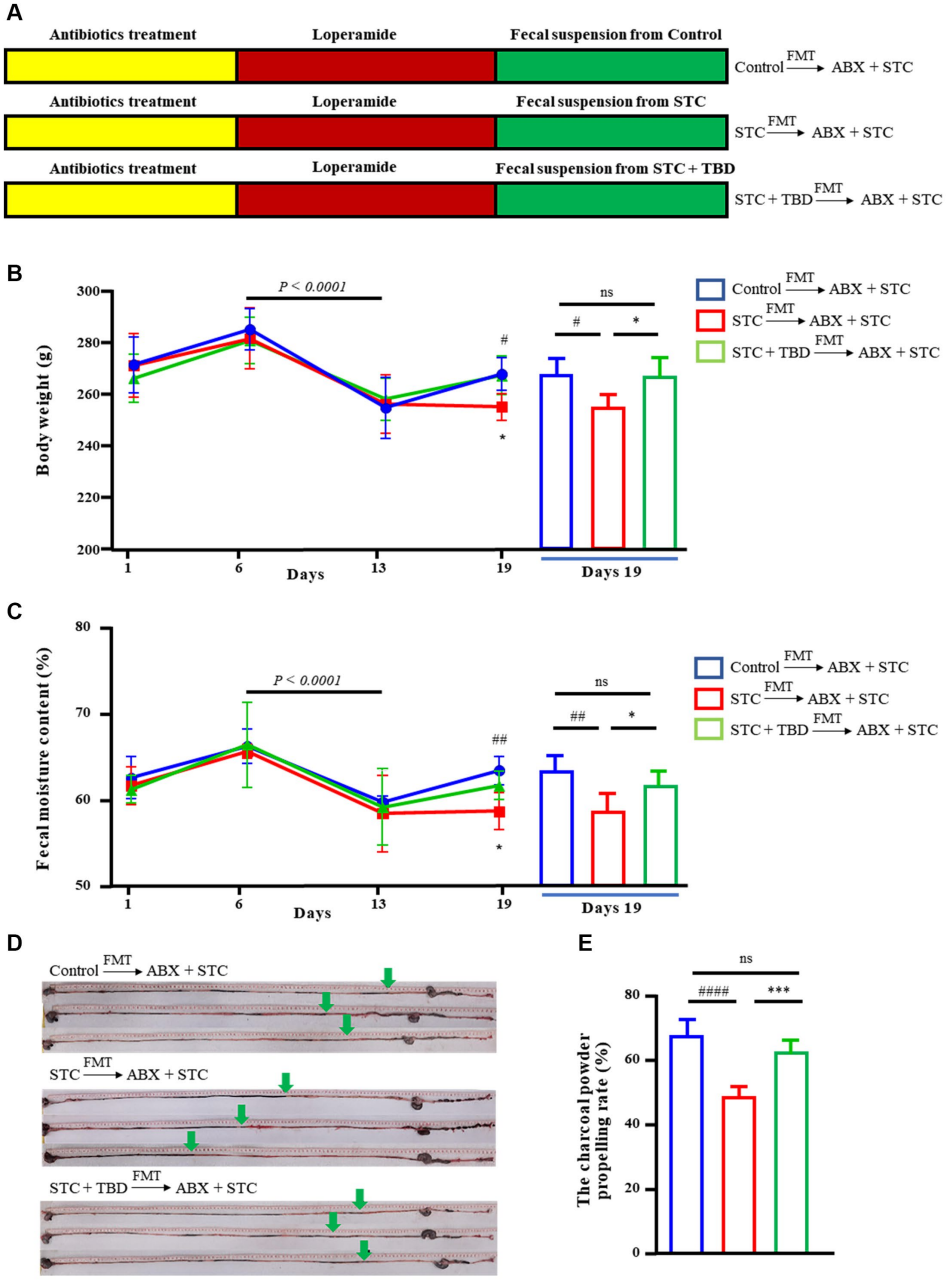
Slow transit constipation is reversed by fecal transplantation from TBD-treated mice to STC rats. **(A)** Design experiment. **(B)** Body weight of the Control → ABX + STC group, the STC → ABX + STC group, and the STC + TBD → ABX + STC group. **(C)** The Fecal moisture content of the Control → ABX + STC group, the STC → ABX + STC group, and the STC + TBD → ABX + STC group. **(D,E)** The charcoal power propelling rate of the Control → ABX + STC group, the STC → ABX + STC group, and the STC + TBD → ABX + STC group. Data were represented as mean ± SD. Control → ABX + STC group: the fecal suspension of the control group was transplanted to the established ABX + STC model group by the FMT method, STC → ABX + STC group: the fecal suspension of the STC group was transplanted to the established ABX + STC model group by the FMT method, STC + TBD → ABX + STC group: the fecal suspension of the STC + TBD group was transplanted to the established ABX + STC model group by the FMT method. ^#^*p* < 0.05, ^##^*p* < 0.01, ^####^*p* < 0.0001 (comparison with Control → ABX + STC group); ^*^p < 0.05, ^**^p < 0.01, ^***^p < 0.001 (comparison with STC → ABX + STC group).

To examine the effect of TBD, an STC rat model was established (ABX + STC) (Figure 3A). The body weight and fecal water content of the rats were significantly reduced on day 13 than on day 6 (*p* < 0.0001) (Figures 3B,C), indicating that the STC model was successfully established. After establishing the model, the fecal suspension of donor microflora was transplanted into recipient rats by FMT. On day 19, compared with the rats in the Control → ABX + STC group, the rats in the STC → ABX + STC group had significantly decreased body weight, fecal water content, and charcoal power propelling rate (Figures 3B–E). Further, compared with the rats in the STC → ABX + STC group, the rats in the STC + TBD → ABX + STC group showed significantly higher body weight, fecal water content, and charcoal power propelling (Figures 3B–E). These results suggested that the fecal suspension from TBD-treated rats could effectively alleviate symptoms of constipation in loperamide-induced STC rats.

### 3.4 TBD fecal transplants alleviate pathological damage to the colon

**Figure 4:**
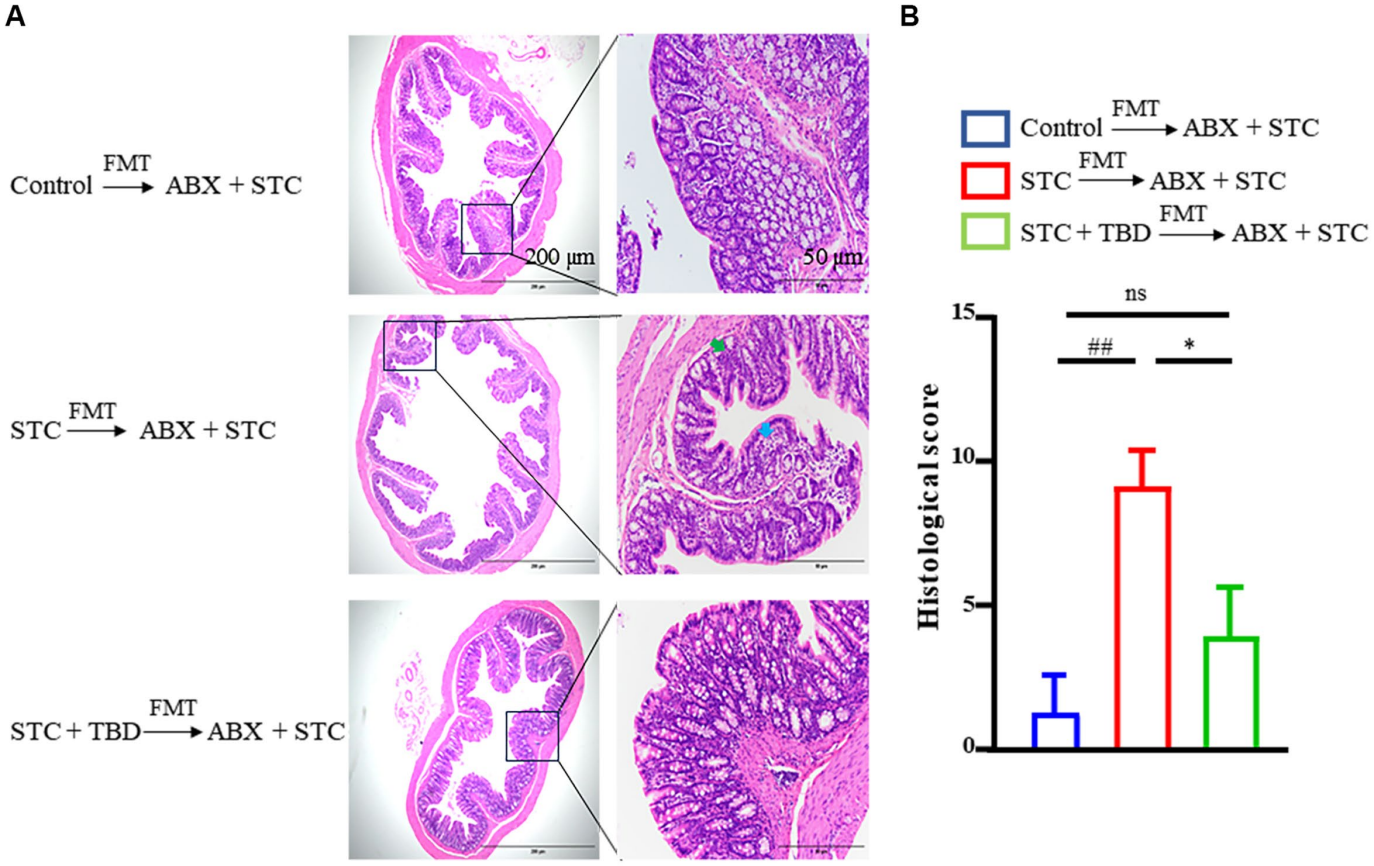
TBD fecal transplants alleviate pathological damage to the colon **(A)** Hematoxylin and eosin (H&E) staining of the colon. The blue arrow indicates gland destruction, the green arrow indicates goblet cell loss. **(B)** Histological score. Control → ABX + STC group: the fecal suspension of the control group was transplanted to the established ABX + STC model group by the FMT method, STC → ABX + STC group: the fecal suspension of the STC group was transplanted to the established ABX + STC model group by the FMT method, STC + TBD → ABX + STC group: the fecal suspension of the STC + TBD group was transplanted to the established ABX + STC model group by the FMT method. ^##^*p* < 0.01 (comparison with Control → ABX + STC group); ^*^p < 0.05 (comparison with STC → ABX + STC group).

In the STC → ABX + STC rats, the intestinal gland in the lamina propria was dissolved and necrotic, and the number of goblet cells in the intestinal gland was significantly reduced. Compared with the rats of the STC → ABX + STC group, the intestinal gland necrosis in the lamina propria of the colon tissue was improved and the goblet cell number in the intestinal gland was significantly increased in the rats of the STC + TBD → ABX + STC group (Figure 4A). Compared with Control → ABX + STC group, the histological score of colonic tissue in STC → ABX + STC group was significantly higher (p < 0.01). Compared with STC → ABX + STC group, the colonic histological score of rats in STC + TBD → ABX + STC group were significantly decreased (*p* < 0.05) (Figure 4B). These results indicated that fecal transplants from TBD-treated rats alleviated pathological damage to the colon.

### 3.5 TBD fecal transplants promote the activation of the 5-HT signaling pathway in STC rats

Next, 5-HT, TPH1, the rate-limiting enzyme for 5-HT synthesis, and 5-HT4R were investigated to further study whether the alleviation of STC by the fecal suspension from TBD-treated rats was related to the 5-HT signaling pathway.

**Figure 5:**
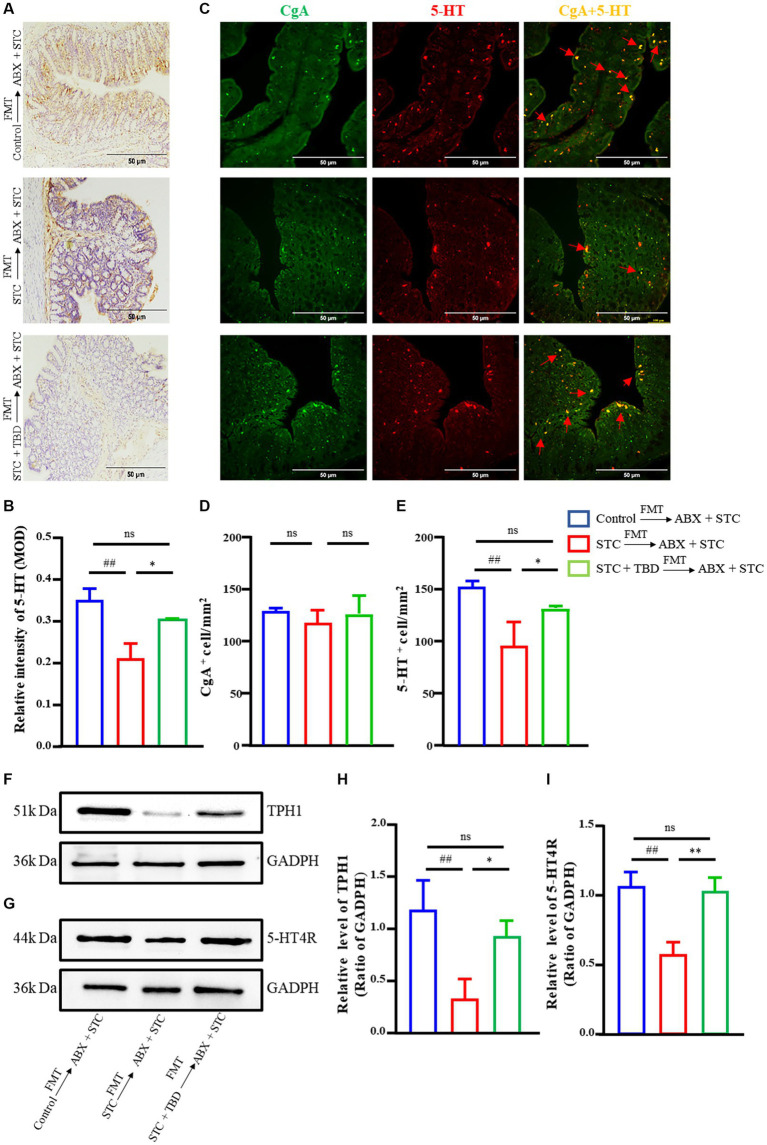
Fecal suspension from the TBD group promoted the secretion of 5-HT and activated its receptor pathway in the STC rats. **(A,B)** Immunohistochemical results of 5-HT in colon tissue. MOD, mean optical density. **(C)** Colonic tissues were immunofluorescence stained with primary antibodies against CgA to mark chromaffin cells (Green) and mark 5-HT (Red). Arrows indicate CgA^+^ positive cells and 5-HT staining. **(D)** Quantitation of CgA^+^ cell number per mm^2^. **(E)** Quantitation of 5-HT^+^ cell numbers per mm^2^. **(F–I)** WB results of TPH1 and 5-HT4R in colon tissue. Data were represented as mean ± SD. Control → ABX + STC group: the fecal suspension of the control group was transplanted to the established ABX + STC model group by the FMT method, STC → ABX + STC group: the fecal suspension of the STC group was transplanted to the established ABX + STC model group by the FMT method, STC + TBD → ABX + STC group: the fecal suspension of the STC + TBD group was transplanted to the established ABX + STC model group by the FMT method. ^##^*p* < 0.01, ^###^*p* < 0.001 (comparison with Control → ABX + STC group); ^*^p < 0.05, ^**^p < 0.01, ^***^p < 0.001 (comparison with STC → ABX + STC group).

We found that following FMT, the expression of 5-HT in STC → ABX + STC rats was lower than that in the Control → ABX + STC rats, whereas the expression of 5-HT in STC + TBD → ABX + STC rats was significantly increased (Figures 5A,B). The expressions of TPH1 and 5-HT4R were decreased in STC → ABX + STC rats compared with those in the Control → ABX + STC rats, and those in STC + TBD → ABX + STC rats were elevated compared with those in STC → ABX + STC rats (Figures 5F–I). These results showed that the fecal suspension of TBD alleviated STC by increasing the secretion of 5-HT and activating its receptor pathway.

To determine whether the fecal suspension of TBD targets enterochromaffin cells (ECs) to promote the synthesis of 5-HT, chromogranin A (CgA) was used to label ECs. Our results showed no significant differences in the number of CgA^+^ cells across the three groups (Figures 5C,D). Compared with the rats in the Control → ABX + STC group, the rats in the STC → ABX + STC group had a significantly reduced number of 5-HT-positive cells/mm^2^ (Figures 5C,E). There was a significant increase in the number of 5-HT-positive cells/mm^2^ in the STC + TBD → ABX + STC group (*p* < 0.05). These results indicate that the fecal suspension from TBD-treated rats regulates intestinal motility by targeting the 5-HT signaling pathway without altering the count of EC cells.

### 3.6 TBD fecal transplants alleviate STC by regulating *Firmicutes* and *Bacteroidota*

**Figure 6:**
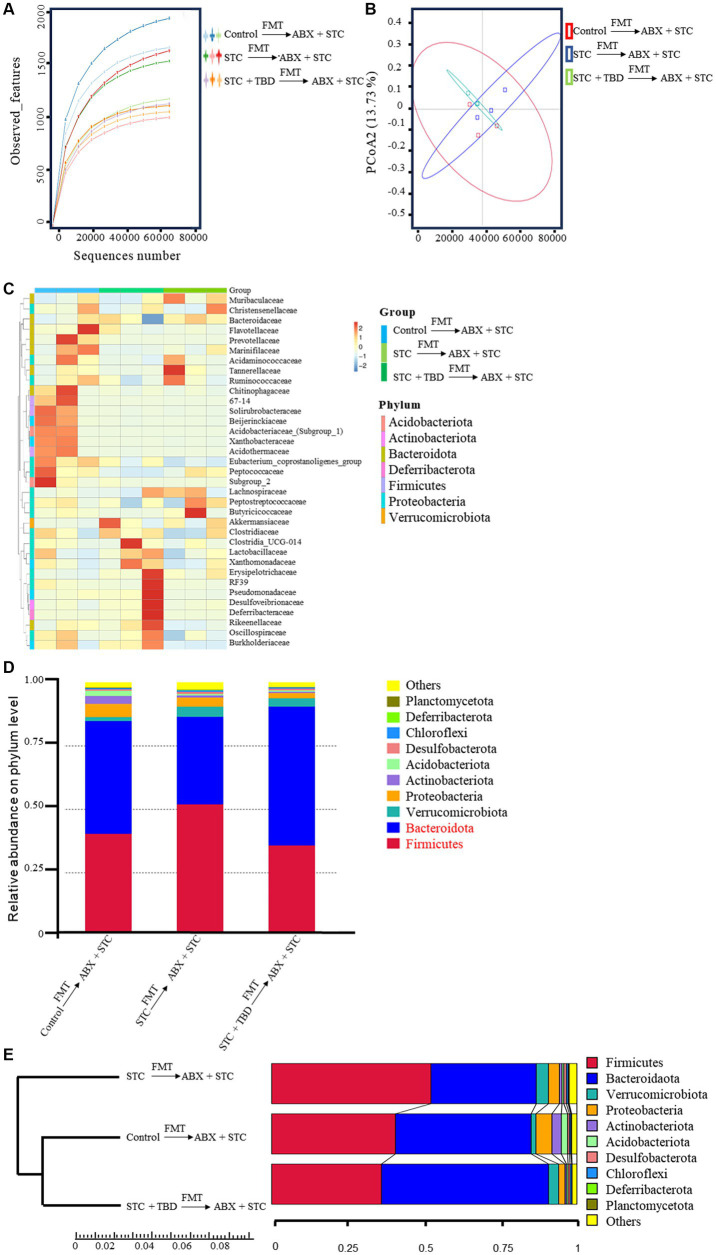
Analysis of gut microbiota following fecal transplantation. **(A)** Rarefaction curve of intestinal bacteria. **(B)** Plots of weighted UniFrac-based PCoA. **(C)** Relative abundance heat map of intestinal bacteria. The ordinate represents the functional annotation, and the abscissa is the sample information. Red and blue represent the higher and lower relative abundances of a function in the groups, respectively. **(D)** Relative abundance of intestinal bacterial communities at phylum. **(E)** The UPGMA cluster tree analysis. Data are expressed as the mean ± SD. Control → ABX + STC group: the fecal suspension of the control group was transplanted to the established ABX + STC model group by the FMT method, STC → ABX + STC group: the fecal suspension of the STC group was transplanted to the established ABX + STC model group by the FMT method, STC + TBD → ABX + STC group: the fecal suspension of the STC + TBD group was transplanted to the established ABX + STC model group by the FMT method.

To confirm that the fecal suspension from TBD-treated rats regulated the intestinal microflora, the intestinal microbiota of rats that underwent FMT was examined. The sparse and rank abundance curves (reflecting the rationality of sequencing data and species richness) indicated that the amount of sequencing data was reasonable, and the species distribution was uniform in this experiment (Figure 6A). Principal coordinate analysis (PCoA) results showed that the STC + TBD → ABX + STC group was close to the Control →ABX + STC group, and the STC → ABX + STC group was far from the Control → ABX + STC group, suggesting that the colon microflora structure of the STC + TBD → ABX + STC group was more similar to that of the Control → ABX + STC group (Figure 6B). Relative abundance thermograms of the top 10 bacteria were plotted. Differences in the bacterial community diversity of each treatment group were obvious in the relative proportion values and color changes on the heat map (Figure 6C). At the phylum level, *Firmicutes* and *Bacteroidota* were the predominant intestinal bacteria. The relative abundance of *Bacteroidota* was decreased and the relative abundance of *Firmicutes* was increased in the STC → ABX + STC rats compared with those in the Control → ABX + STC rats (Figure 6D). Moreover, the relative abundance of *Bacteroidota* was increased and the relative abundance of *Firmicutes* was decreased in the STC + TBD → ABX + STC rats compared with those in the STC → ABX + STC rats (Figure 6D). To investigate specific alterations in the gut microbiota, we conducted a UPGMA cluster analysis to compare the major relative abundances at the phylum level among the different groups (Figure 6E). Compared with the Control → ABX + STC group, the STC → ABX + STC group exhibited a substantial dysbiosis in the ratio of *Firmicutes* to *Bacteroidota*: *Firmicutes* abundance significantly was increased, whereas *Bacteroidota* abundance was notably decreased. The STC + TBD → ABX + STC group adjusted the ratio of these two bacteria to achieve a gut microbiota structure similar to that under normal conditions (Figure 6E). These results show that a fecal suspension of TBD can change the structure of the intestinal microbiota, particularly, increase the abundance of *Bacteroidota* and decrease the abundance of *Firmicutes*.

## 4 Discussion

**Figure 7:**
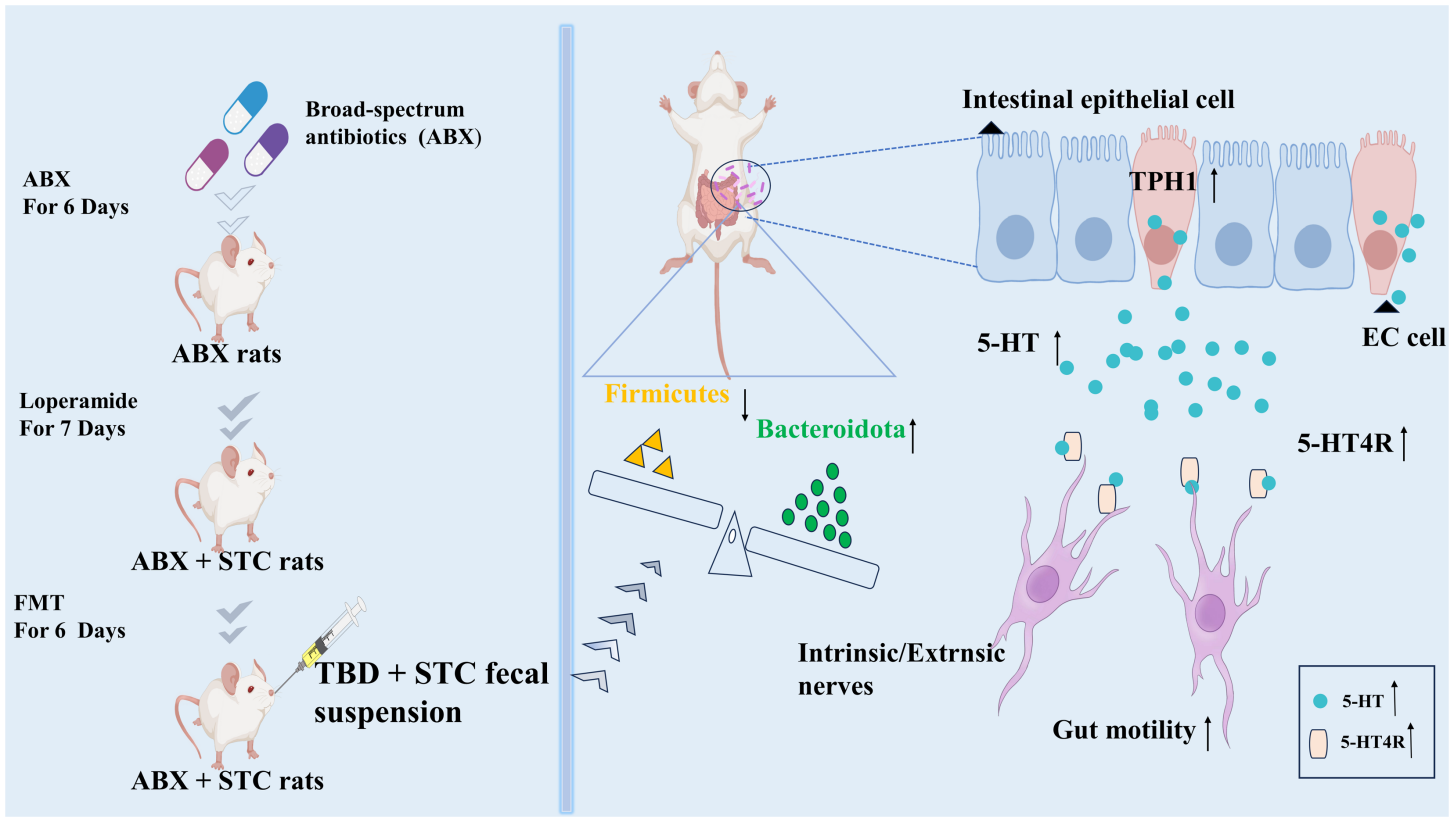
TBD fecal suspension alleviates STC by activating the 5-HT signal and changing the structure of intestinal microbiota.

STC is a common digestive disease characterized by difficulty in defecation. TBD mainly contains kaempferol, quercetin, luteolin, naringenin, (+)-catechin and neohesperidin active ingredients. These suggested that the regulating effect of tongbian decoction on 5-HT maybe related to the above active components (Qin et al., 2019; Liu et al., 2020; Josiah et al., 2022; Lee et al., 2022; Wang et al., 2023; Zhang et al., 2023). We also confirmed that TBD-treated STC rats mainly activated the 5-HT signaling pathway and changed the structure of the intestinal microbiota. Our study showed that the fecal suspension obtained following TBD treatment could alleviate STC symptoms, which suggests that the intestinal microbiota may be a key target of TBD in alleviating STC (Figure 7).

Studies have shown that intestinal microbiota plays an important role in intestinal motility and potentially influences the efficacy of anti-constipation drugs (Yano et al., 2015). We used broad-spectrum ABX to generate pseudo-germ-free rats and found that intestinal motility was accelerated in pseudo-sterile rats, which is consistent with the findings of Lijun (2021). Conversely, Wichmann et al. (2013) found that the same ABX decreased gut motility in mice. To account for these varying results, we considered species differences between rats and mice, as well as variations in the duration of drug administration and rearing environment, as potential factors. We also conducted a gut microbiota analysis in pseudo-germ-free rats, which revealed that the abundance and alpha diversity of the gut microbiota were significantly decreased in the ABX + group. Additionally, specific microbial analysis identified *Pseudomonas* as the predominant species after the administration of broad-spectrum ABX.

ECs differentiate from intestinal epithelial basal stem cells (Gershon and Tack, 2007) and convert tryptophan to 5-HT through the action of TPH1, producing 95% of the 5-HT in the body (Reigstad et al., 2015). Alterations in EC numbers and concentration of 5-HT are closely related to various gastrointestinal diseases (Kim et al., 2010; Sia et al., 2013); notably, the EC-TPH1-5-HT signaling pathway plays a key role in the development of constipation. Additionally, 5-HT is primarily distributed in the gastrointestinal tract, and its effects are mediated mainly through 5-HT receptors (Gershon and Tack, 2007; Bockaert et al., 2011). The 5-HT4R, which is one of the 5-HT receptors, is closely related to gastrointestinal motility, and its decreased expression affects intestinal peristalsis (Tonini, 2005; Kim, 2009). The results in this study showed that TBD treated rats-derived fecal suspension can increase the level of TPH1 and 5-HT in the intestine of STC rats. We also found that the number of CgA^+^ positive cells in the intestines of rats administered fecal suspension obtained following TBD treatment showed an increasing trend but there was no statistical difference, which is consistent with the findings of Cao et al. (2017) and Yano et al. (2015). These findings collectively suggest that the fecal suspension obtained following TBD treatment regulates intestinal motility by targeting the 5-HT signaling pathway without affecting the count of EC cells.

Dysbiosis of intestinal microbiota represents a major characteristic of patients with constipation (Zhuang et al., 2019). The intestinal microflora of patients with STC is abnormal in terms of quantity and composition. Recent studies have shown a decrease in the relative abundance of *Bacteroidota* and an increase in the relative abundance of *Firmicutes* in patients with STC (Tian et al., 2020), which is consistent with our findings in SCT rats. Furthermore, our 16S rRNA analysis indicated that the fecal suspension from TBD-treated rats increased the number of *Bacteroidota* (beneficial bacteria) and inhibited *Firmicutes* (harmful bacteria) in the intestine, thus affecting the composition and quantity of intestinal microorganisms to alleviate STC.

Our study also revealed that antibiotics can lead to increased gut motility, a phenomenon similarly observed following administration of fecal suspensions derived from TBD-treated rats. However, the underlying mechanisms for this enhanced intestinal activity require further investigation. Our analysis showed that the specific microbiota in the rats was *Pseudomonas* after the administration of antibiotics. A previous study has suggested that *Pseudomonas aeruginosa* is associated with antibiotic-related diarrhea (Kim et al., 2001) *Pseudomonas aeruginosa* and *Pseudomonas* belong to the same genus and may be one of the factors causing an increase in intestinal peristalsis in rats. In contrast, the number of *Bacteroidota* increased after administration of the fecal solution obtained from TBD-treated rats. A previous report suggests that *Bacteroidota* are beneficial bacteria that promote gastrointestinal peristalsis (Hongliang, 2017). These observations underscore the differing impacts on gut microbiota by antibiotics and fecal microbiota from TBD-treated animals.

Collectively, the results in this study emplasize that the stability of the gut microbiota is crucial for optimal gastrointestinal motility. TBD, a traditional Chinese medicine, can alleviate STC by restoring gut microbiota stability.

## 5 Conclusion

Our study showed that TBD improved constipation in STC rats by regulating the relative abundances of *Firmicutes* and *Bacteroidota*. The present study provides new evidence for the therapeutic effect of TBD from the perspective of intestinal microflora and 5-TH signaling. In addition, intestinal microbiota can affect the changes of metabolites, such as bile acid, short-chain fatty acids, and tryptophan metabolism. It has been reported that the metabolites of intestinal microbiota affect the synthesis of 5-HT, thus promoting gastrointestinal motility (Yano et al., 2015; Zhan et al., 2022). To further elucidate the direct mechanisms involved, future work should focus on identifying which specific metabolites are influenced by the fecal suspensions derived from TBD treatment.

The limitation of this study is that only male rats were selected in the experiment, and clinically constipation is not limited to male patients. For this reason, our study may cannot fully represent the entire clinical pathogenesis of STC. However, the purpose of this study was to elucidate the mechanism of action of TBD targeting the microbiome to treat STC. Therefore, the possible effects of hormones on STC development in female rats need to be excluded in experimental design.

Our study promotes the clinical application of traditional Chinese medicine and provides a new strategy for the treatment of slow transit constipation.

## Data availability statement

The data presented in this study are deposited in the NCBI Sequence Read Archive (SRA) under the Bio project number: PRJNA1010002.

## Ethics statement

The animal study was approved by the Laboratory Animal Ethics Committee of Nanjing Hospital Affiliated with Nanjing Medical University (approval number: DWSY-22113242). The study was conducted in accordance with the local legislation and institutional requirements.

## Author contributions

HL: Data curation, Writing – original draft. NL: Writing – original draft. DL: Methodology, Writing – original draft. YQ: Writing – review & editing. XS: Methodology, Writing – original draft. YH: Writing – original draft. YW: Writing – review & editing. XH: Supervision, Writing – review & editing. TX: Supervision, Writing – review & editing.
